# Multifaceted Interpretation of Colon Cancer Stem Cells

**DOI:** 10.3390/ijms18071446

**Published:** 2017-07-05

**Authors:** Yuichiro Hatano, Shinya Fukuda, Kenji Hisamatsu, Akihiro Hirata, Akira Hara, Hiroyuki Tomita

**Affiliations:** 1 Department of Tumor Pathology, Gifu University Graduate School of Medicine, Gifu 501-1194, Japan; yuha@gifu-u.ac.jp (Y.H.); u2001087@edu.gifu-u.ac.jp (S.F.); y3f3f84d72xsx@yahoo.co.jp (K.H.); ahara@gifu-u.ac.jp (A.H.); 2 Division of Animal Experiment, Life Science Research Center, Gifu University, 1-1 Yanagido, Gifu 501-1194, Japan; akatsuki@gifu-u.ac.jp

**Keywords:** colon cancer, cancer stem cell, intestinal stem cell, genetics, epigenetics, bidirectional conversion, tumor heterogeneity

## Abstract

Colon cancer is one of the leading causes of cancer-related deaths worldwide, despite recent advances in clinical oncology. Accumulating evidence sheds light on the existence of cancer stem cells and their role in conferring therapeutic resistance. Cancer stem cells are a minor fraction of cancer cells, which enable tumor heterogeneity and initiate tumor formation. In addition, these cells are resistant to various cytotoxic factors. Therefore, elimination of cancer stem cells is difficult but essential to cure the malignant foci completely. Herein, we review the recent evidence for intestinal stem cells and colon cancer stem cells, methods to detect the tumor-initiating cells, and clinical significance of cancer stem cell markers. We also describe the emerging problems of cancer stem cell theory, including bidirectional conversion and intertumoral heterogeneity of stem cell phenotype.

## 1. Cancer Stem Cells (CSCs) and Colon Cancer

Cancer stem cell (CSC) theory is the concept that cancer tissue contains a minor population of cells with stem cell properties [1]. This minor fraction is believed to contribute to the development and expansion of cancerous tissues, similar to tissue stem cells that generate normal functional histological units, tissues, and organs. Therefore, the minimal qualifications for CSCs include self-renewal and tumor formation ability. Self-renewal is essential to maintain their stemness, which is the potential for both cellular proliferation and differentiation. By asymmetric cell division, CSCs are long-lived, have a low proliferative nature, and simultaneously produce actively proliferating cancer progenitor cells. The long-lived, low proliferative CSCs are resistant to cytotoxic conditions, whereas the actively proliferating cancer progenitor cells have an advantage of tumor propagation. Together, these heterogeneous components lead to tumor formation, which results in multiple and recurrent lesions in the clinical course of the disease. The concept of CSCs is a sophisticated interpretation of intratumoral heterogeneity.

Although the idea of CSCs originally occurred about 160 years ago [2], the first demonstration was made in 1997. Bonnet and Dick reported that CD44^+^/CD38^−^ leukemia cells possessed CSC properties, i.e., self-renewal, potential for proliferation and differentiation, and tumor formation ability, in nude mice [3]. After the first report, tumor-initiating cells with a stem cell phenotype were identified in several types of solid tumor, including colon cancer [4]. Currently, CSC theory provides a universal basis to understand tumor cell biology.

**Figure 1:**
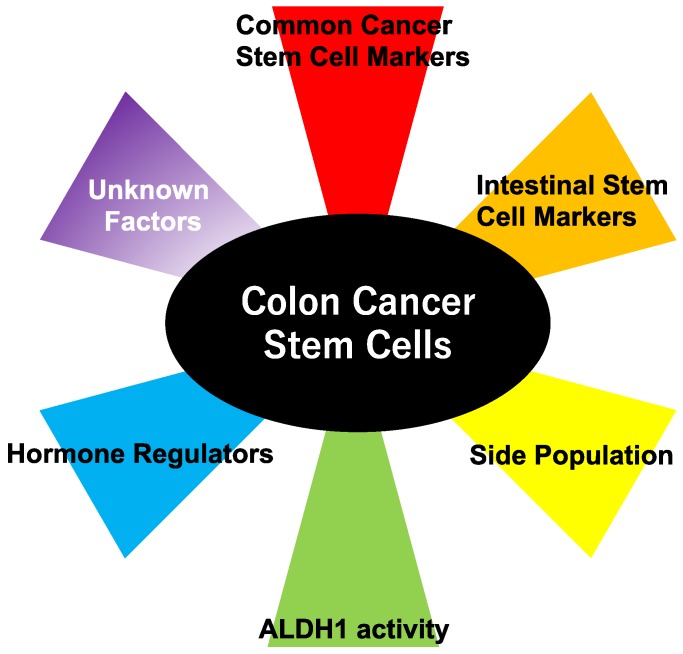
Various methods to detect colon cancer stem cells. Colon cancer stem cells are detectable on the basis of common cancer stem cell markers (Section 2.1), intestinal stem cell markers (Section 2.2 and Section 2.3), side population (Section 2.5), aldehyde dehydrogenase (ALDH1) activity (Section 2.6), and hormone regulators (Section 2.7). Considering the multifaceted nature of cancer stem cells, there must be unknown factors that mark cancer stem cells (for example, epithelial–mesenchymal transition (EMT) inducers, Section 2.4).

Colon cancer is the fourth most deadly cancer in the world [5]. The incidence is rising in many countries, potentially because of widespread adoption of the Western diet and lifestyle. Worldwide mortality of colon cancer is approximately 50%, which varies according to the available therapeutic options. Interestingly, a systematic review reveals that surgical debulking of liver metastasis improves the prognosis of patients with colorectal cancer [6]. Although this macroscopic intervention is efficacious in advanced stage colon cancer, new clinical approaches to target CSCs are desired to eliminate viable cancer cells completely. The following sections describe a strategy to detect colon cancer cells with a stem cell-like/tumor-initiating phenotype (Figure 1).

## 2. Methods to Detect Colon Cancer Stem Cells

### 2.1. Cell Surface CSC Markers

Cell surface proteins have contributed to the isolation and identification of tumor-initiating cancer cells [7]. Based on the presence of specific cell surface proteins, flow cytometric analysis divides cancer cells into two populations. The isolated populations are often tested for their stem cell properties using in vitro assays, and injected into nude mice to investigate their tumor forming ability. These experiments are often attempted to demonstrate whether the cell surface protein of interest is a CSC marker. Not surprisingly, the surface CSC markers identified in past studies are concomitantly expressed in the CSC population. Therefore, dual color flow cytometric analysis with combined CSC cell markers yields a highly precise CSC population. The following sections introduce selected cell surface proteins that function as candidate CSC markers in colon cancer.

#### 2.1.1. CD44

Of the first CSC phenotype, CD44^+^/CD38^−^ [3], CD44 positivity in CSCs is found not only in leukemia but also other solid tumors [8]. CD44 is a multifunctional cell surface adhesion protein that reacts with extracellular matrix components, i.e., hyaluronan. The CD44 protein family consists of various isoforms created by alternative splicing of ten different variant exons. One CD44 variant associated with cancer metastasis was first discovered in a rat carcinoma cell line [9]. Subsequently, CD44 expression in colon cancer was reported [10] before the demonstration of leukemia stem cells. Expression of CD44 v6 is frequently found in advanced stages of colon cancer [11] and is associated with poorer prognosis [12].

CD44 v6 binds hepatocyte growth factor (HGF) and vascular endothelial growth factor (VEGF) [13,14]. These interactions are important to Ras activation and angiogenesis in cancer [15,16]. Subsequent alterations of cellular signaling and the microenvironment contribute to the tumor initiating ability of cancer cells. Interestingly, the expression of CD44 v6 is inducible in colon cancer cells by Rho kinase (ROCK) inhibitors or a myosin II inhibitor [17]. Increased CD44 expression leads to enhancement of CSC properties in colon cancer cells. These findings support the interconversion of cancer stemness: CSCs differentiate into non-CSCs while non-CSCs give rise to CSCs through the reactivation of stem cell markers.

#### 2.1.2. CD133

CD133, also known as prominin-1, is a 120 kDa five transmembrane cell surface protein that affects multicellular functions including stemness, tumorigenesis, chemo-/radio-resistance, metabolism, autophagy, and apoptosis [18]. The expression of CD133 in colon cancer is one of the most important features of colon cancer stem cells. CD133^+^ human colon cancer cells are able to initiate tumor formation in immunodeficient mice [4]. Todaro et al. reported that CD133^+^ cells, approximately 2% of human colon cancer cells, are treatable by an interleukin-4 inhibitor [19]. In addition, flow cytometric analysis revealed that CD133^+^ colon cancer cells overlap the cell fractions expressing CD44, CD29, CD24, and CD166 [20]. Isolated single CD133^+^ colon cancer cells show self-renewal and multi-lineage differentiation. Consistent with this evidence, transgenic mice demonstrate that CD133 marks intestinal stem cells, which transform into neoplastic cells by expression of mutated β-catenin [21]. Recent evidence suggests that CD133 is expressed in both colon CSCs and non-CSCs [22]. However, the AC133 epitope reacts with the most frequently used anti-CD133 antibody and is detected in CSCs, probably due to differential glycosylation [23]. The most precise marker of CSCs is the AC133 epitope, not the CD133 protein. Thus, there are considerable discrepancies in available research results, based on the difference between these markers.

#### 2.1.3. CD24

CD24, also known as heat stable antigen, is a B cell-related short glycoprotein whose expression is associated with several types of cancer. The first report described this surface protein as a CSC marker in breast cancer [24]. CD24^−/low^ cancer cell subpopulations with the CD44^+^ and/or ESA^+^ phenotype have higher tumorigenic abilities compared to the CD24 positive counterpart. In contrast, positivity for CD24 is a CSC marker of ovarian and pancreatic cancer [25,26]. In colon cancer, clinical research studies report that expression of CD24 is associated with differentiation. Using immunohistochemical analysis of CD24 in colon cancer, two papers described contradictory significance of CD24 expression [27,28]. These results suggest that CD24 plays a different role in each cancer type. To unveil the detailed mechanism of CD24 function in cancer, further investigation is necessary.

#### 2.1.4. CD29

CD29, also known as integrin β1, is the most plentiful β subunit of integrin, a cell surface receptor that assists adherence to the extracellular matrix, i.e., collagen, fibronectin, and laminin [29]. Integrin, which is an αβ heterodimer, is a structural linker between the cytoskeleton and extracellular matrix, as well as a mediator of transmembrane signaling. Various integrins are involved in diverse cellular processes such as adhesion, development, inflammation, and hemostasis.

High expression of CD29 is observed in normal stem cells in various organs. In colon, CD29 is highly expressed in the lower part of the crypt, where stem and progenitor cells reside [30]. Conditional deletion of CD29 leads to hyperplasia and dysplasia in the intestinal epithelium, which is associated with increased TCF4 signaling and decreased hedgehog signaling [31]. This suggests that CD29 is a key regulator of intestinal proliferation and maintenance by modulating specific signal transduction pathways. In addition, CD29 is gathering attention as a CSC marker that is related to chemoresistance and metastasis, especially in breast cancer [32]. However, the significance of CD29 in colon cancer has yet to be elucidated.

#### 2.1.5. CD26

CD26, also known as dipeptidyl peptidase IV, is a multifunctional transmembrane protein with serine protease activity [33] that is a new drug target for type 2 diabetes [34]. Recently, Pang et al. demonstrated that the CD26^+^ subpopulation of colon cancer cells was able to both initiate tumor formation and metastasize to liver [35]. CD26 knockdown in CD26^+^ cells weakened their invasive, adherence, migration, and tumor formation abilities. This alteration was associated with the decreased expression of epithelial–mesenchymal transition (EMT)-related factors and phosphorylated integrin β1(CD29), and was consistent with a previous report [36]. Of note, administration of chemotherapeutic agents reduced the tumor volume and enrichment of the CD26^+^ subpopulation in SCID mice. Taken together, CD26 is a possible CSC marker to induce the EMT, and may collaborate with another CSC marker, CD29. Consistent with these findings, clinical research has reported that expression of CD26 is related to a poor prognosis in colon cancer [37,38,39].

#### 2.1.6. CD166

CD166, also known as activated leukocyte cell adhesion molecule, is an immunoglobulin superfamily member that is a ligand of CD6, a marker of T cells and thymocytes [40]. This surface protein is associated with T cell activation as its name suggests, but its potential usefulness as a CSC marker is also attracting interest. Dalerba et al. found that CD166 was an additional differentially expressed marker for CSC isolation in colon cancer [41]. Further investigation revealed that CD166 was expressed in mouse and human intestinal stem cells, and human colon adenocarcinoma [42]. Interestingly, some colon cancer cells showed not only cytomembranous but also cytoplasmic positivity of CD166. Although meta-analyses of CD166 expression in colon cancer studies resulted in little clinical significance [43,44], further study is needed to consider these distinctive expression patterns.

#### 2.1.7. CD326 

CD326, also known as an epithelial cell adhesion molecule, was first discovered as a specific antigen for human colon carcinoma [45]. This transmembrane protein affects multiple cellular processes, including proliferation, differentiation, and cell death, and is widely expressed in several types of normal/benign and malignant epithelial cells [46].

Immunohistochemical analyses clarified that the expression of CD326 was associated with normal intestinal epithelium [47], and enhanced in malignancy [48]. Another immunohistochemical analysis estimated that CD326 expression in colon cancer occurred at a strong (>70% of tumor cells) and high frequency (almost all cases of conventional human colon cancer) [49], suggesting that the single marker of CD326 in colon cancer is inappropriate to capture the “minor” population of tumor-initiating cells. However, recent evidence suggests that CD326 is a marker of the CSC phenotype [50]. In hepatocellular carcinoma, CD326 is activated by canonical Wnt signaling and vice versa [51]. Considering the importance of canonical Wnt signaling in both intestinal stemness and colon carcinogenesis, the functional significance of CD326 in colon cancer should be investigated.

### 2.2. An Active Intestinal Stem Cell Marker, Leucine-Rich Repeat-Containing G-Protein-Coupled Receptor 5 (Lgr5)

Examination of intestinal crypts is one of the most active research subjects of stem cell biology. Intestinal stem cells are thought to be active tissue stem cells that promote rapid turnover of intestinal epithelia to wash out cytotoxic factors from the daily diet. Cheng and Leblond identified intestinal epithelial stem cells, which are also known as crypt base columnar cells (CBCs), in the bottom of mouse small intestinal crypts by injecting ^3^H-thymidine [52]. This radiolabel pulse-chase experiment clearly demonstrated that CBCs give rise to multiple types of differentiated cells by asymmetric cell division.

*Lgr5* is the most established marker of active intestinal stem cells. Barkers and colleagues selected *Lgr5* for analysis [53]. *Lgr5* is a downstream target of the canonical Wnt pathway and appears to play an important role in maintaining stemness in the intestinal crypt. Consistent with the hypothesis, a transgenic mouse study demonstrated that expression of *Lgr5* was confined to CBCs, which have abilities of self-renewal and multipotency to differentiate. Although LGR5 was previously recognized as an orphan receptor, it is now recognized as a Wnt enhancer that binds R-spondins [54]. Based on the function of *Lgr5* to enhance the canonical Wnt pathway, it is reasonable that LGR5 expression in intestinal stem cells leads to the formation of an automatic amplification circuit to maintain their stemness. Additional studies reported that isolated intestinal cells expressing *Lgr5* show stem cell properties, and a single cell was able to build intestinal organoids in 3D culture conditions [55]. Collectively, *Lgr5* is a definitive intestinal stem cell marker that governs the canonical Wnt pathway.

A relationship between *Lgr5* expression and intestinal tumorigenesis has been reported. Wnt activation by an *APC*/β-catenin mutation is the initial step for intestinal tumorigenesis, which is also known as the adenoma-carcinoma sequence [56]. Conditional deletion of *Apc* leads to cellular transformation of not only stem cells but also progenitor cells in mice [57]. However, *Ah-Cre* expressing non-stem intestinal cells are able to transform into dysplastic cells, but most of the lesions fail to develop into intestinal neoplasia. In contrast, LGR5-GFP^+^ stem cells efficiently form adenomatous lesions with high expression of β-catenin and LGR5-GFP. This lineage tracing study suggests that active intestinal stem cells are suitable for originating intestinal tumor cells.

Further analysis of microadenomas elucidated that LGR5-expressing cells are mixed with Paneth cells which are a stem cell niche in intestinal crypts. This suggests that a microenvironment like normal intestinal crypts is necessary in the early stage of intestinal tumorigenesis [58]. In addition, a model simulating an adenoma-carcinoma sequence has been reported using cell culture of intestinal organoids [59,60]. These findings support a “bottom-up” model of intestinal carcinogenesis [61]. However, counterevidence that indicates a “top-down” model also exists [62]. Schwitalla and collaborators suggested that LGR5^−^ intestinal cells have cell plasticity, which enabled them to dedifferentiate into LGR5^+^ stem cells and give rise to tumor-initiating cells through Wnt activation mediated by NF-κB signaling [63].

### 2.3. Quiescent Intestinal Stem Cell Markers

Another fraction of intestinal stem cells is located at the +4 position counting Paneth cell nuclei from the crypt bottom. The +4 position, which occurs directly above Paneth cells, contains DNA label-retaining cells, suggesting that these minor cells are long-lived and quiescent in nature [64]. Buczacki et al. concluded that the intestinal label-retaining cells are secretory precursor cells arising from LGR5-expressing stem cells, and give rise to LGR5-expressing cells for crypt regeneration and homeostasis after severe injury [65].

*Bmi1* (B lymphoma Mo-MLV insertion region 1, also known as polycomb group RING finger protein 4 or RING finger protein 51) was first identified in mouse lymphomagenesis [66]. *Bmi1*, a member of the polycomb group of genes, regulates proliferative activity in normal and cancer stem cells [67], and the multipotency of hematopoietic stem cells and multipotent progenitor cells, to suppress transcriptional regulators for lineage differentiation [68]. In the intestine, expression of Bmi1 corresponds to the +4 position of crypt cells. A study with transgenic mice demonstrated that Bmi1^+^ cells play a pivotal role in intestinal homeostasis and tumorigenesis [69]. Of note, *Bmi1^+^* cells, as well as label-retaining cells, give rise to *Lgr5^+^* cells and maintain intestinal crypts after artificial ablation of *Lgr5*-expressing cells [70]. This suggests that BMI1^+^ cells are a stem cell source held in reserve in case of critical damage of intestinal crypts.

Similarly, expression of BMI1 is important for the tumor-initiating and self-renewal abilities of human colon cancer cells. Downregulation of BMI1 inhibits tumor cell growth, which is associated with a reduced fraction of tumor-initiating cells [71]. This suggests that the functional significance of *BMI1* is maintenance of stem cell properties in colon cancer cells. Consistent with this notion, clinical studies report that BMI1 expression is a negative predictor in colon cancer [72,73,74,75].

Other quiescent stem cell markers such as homeodomain-only protein (HOPX) [76], doublecortin-like kinase 1 (DCLK1) [77], telomerase reverse transcriptase (TERT) [78], and leucine-rich repeats and immunoglobulin-like domains protein 1 (LRIG1) [79] are associated with colon tumorigenesis, but their detailed function and clinical significance remain unclear.

### 2.4. CSC Markers of Migration

Brabletz et al. proposed the migrating cancer stem cell (MCSC) concept that describes metastasis, which is the final step in the malignant process and the major cause of cancer patient mortality [80]. MCSCs have not only stem cell characteristics but also a migratory phenotype that is induced by the EMT [81]. The EMT, and the reverse conversion, mesenchymal-epithelial transition, play essential roles in embryonic development, tissue homeostasis, tissue recovery, and carcinogenesis. In cancer, the EMT is observed at the boundary between tumor and non-neoplastic tissues, indicating that acquisition of metastatic ability requires a specific microenvironment in addition to internal aberrant cell signaling. Cancer cells in the invasive front show strong nuclear β-catenin expression which activates EMT-related genes through Wnt signaling [82]. Furthermore, extracellular matrix and secreted microenvironment factors also induce the EMT in these cancer cells [83,84].

Probable candidate markers for MCSCs include EMT inducers in addition to some of the cell surface CSC markers. EMT inducers prompt the loss of E-cadherin, which is the initial step in transformation from the epithelial to mesenchymal phenotype [85]. However, it is difficult to differentiate MCSC markers from EMT inducers because of the complex mechanism underlying the EMT. Further investigation is needed before clinical application of these EMT-related factors in colon cancer, as a previous systematic review recommended [86].

### 2.5. Side Population (SP) Cells

One method to identify CSCs is detection of the side population (SP), which is a minor population of cells with the ability to extrude the DNA binding dye, Hoechst 33342. This exporting ability is due to ATP-binding cassette (ABC) transporters that contribute to resistance to cytotoxic agents. Indeed, these transporters are found in many kinds of stem cells. Similarly, SP cells, which are detected in several types of human malignant tumors, generate both SP and non-SP cells and expel cytotoxic agents [87]. In human colon cancer cell lines, SP cells constitute 0.4% of total cancer cells [88] and exhibit resistance to chemotherapeutic agents [89].

An alternative method to identify the SP cell-like phenotype is the detection of ABC transporters. Some ABC transporters are induced in the hypoxic condition and play an important role in the survival of cancer cells exposed to such an unfavorable microenvironment. Hypoxic cancer cells show resistance to not only low oxygen concentrations, but also radiation and chemotherapy [90]. In addition to their drug efflux ability, ABC transporters are associated with other CSC properties including apoptosis, proliferation, differentiation, and cell migration [91]. Therefore, the microenvironment is a potent modifier of the cancer cell phenotype, which leads to intratumoral heterogeneity.

In the ABC transporter family, ABCG2 (also known as BCRP or MXR), ABCC1 (also known as MRP1), and ABCB1 (also known as MDR1 or P-glycoprotein) are important in the efflux of anticancer drugs. Immunohistochemical analysis revealed that expression of ABCC1 and ABCB1 is increased in colonic adenomas [92]. This suggests that the increased expression induces contradictory oncogenic effects in adenoma cells, i.e., both increasing their survival activity and decreasing the potential for additional mutations that would augment tumor progression. In colon cancer, expression of the ABC transporters is related to drug resistance, which can be overcome by small interfering RNAs or inhibitors of these pumps. Although many ABC transporter inhibitors are known to sensitize cancer cells to chemotherapeutic agents in vitro, most clinical trials have failed to demonstrate any clinical benefit [93].

### 2.6. Aldehyde Dehydrogenase (ALDH) 1

ALDH1 is a member of the ALDH gene family that catalyzes the oxidation of aldehydes, including acetaldehyde and retinal (retinaldehyde) (detailed in [94]). Briefly, ALDH1, which is expressed in various normal stem and/or progenitor cells, contributes to cell proliferation and differentiation through activation of retinoid signaling. In addition, ALDH1 has antioxidant properties to reduce reactive oxygen species arising from aldehydes. This detoxifying ability also applies to other cytotoxic factors.

High activity of ALDH1 has been identified as a CSC marker in various types of cancer, including colon cancer [41,95]. The expression of ALDH1 is detectable by an enzymatic assay [96] in addition to the conventional antibody-mediated method. However, this enzymatic assay is problematic in terms of its specificity for different ALDH isoforms and misidentification of similar fluorescent signals. Nevertheless, understanding the functional role of ALDH1 in cancer is essential to develop new diagnostic and therapeutic methods in clinical oncology.

### 2.7. Type III Deiodinase (D3)

Previous reports suggested that thyroid status influences the malignant process. An active form of thyroid hormones, T3, is a transcription factor that controls cell metabolism, proliferation, differentiation, and apoptosis in several tissues [97]. Catalano and coworkers clarified the effect of thyroid hormones in colon CSCs [98]. D3, which is a chief thyroid hormone inactivating enzyme, is strongly expressed in CD133^+^ Wnt^high^ colon cancer cells. In these cells, T3 signaling results in BMP4-mediated cellular differentiation while knockdown of D3 inhibits tumor formation. In addition, T3 treatment removes the chemoresistance of CSCs. These finding suggest that cancer progression is due to not only local mutational events but also the general physiological state of the patient.

## 3. Stem Cell Phenotype in Colon Cancer: Emerging Evidence for Intertumoral Heterogeneity

Recent evidence has indicated that the stem cell phenotype in cancer applies not only to intratumoral heterogeneity but also intertumoral heterogeneity. Indeed, a comprehensive analysis revealed that genomic and epigenetic alterations of colon cancer are both common and diverse [99]. Moreover, the process of these alterations is not always accountable by the adenoma-carcinoma sequence, suggesting that there must be intertumoral heterogeneity in colon cancer [100]. Because the degree of stem cell marker expression varies with each colon cancer, this variation affects the biological behavior of cancer cells.

Consistent with this notion, the intestinal stem cell signature predicts disease relapse of colon cancer [101]. However, low expression of Wnt target genes, including *Lgr5* and *Ascl2*, indicates a more immature phenotype and worse prognosis in colon cancer compared with high Wnt groups [102]. Interestingly, DNA methylation in these immature cells was found in the promoter of these genes. This finding suggests that silencing of intestinal stem cell markers is an important step in colon carcinogenesis and that some intestinal stem cell markers maybe inappropriate as definitive CSC markers. It is also noted that colon cancer stem cells occur in a different context from normal intestinal epithelial cells.

Recently, a new colorectal cancer classification system was proposed based on the cellular phenotype analyzed by gene expression profiling [103]. Of the six classification subtypes, the stem-like subgroup exhibited a good response to chemotherapy, whereas the goblet cell subgroup showed a poor response. Consistent with the evidence, our in vitro experiment clarified that a demethylating agent was efficacious against colon cancer cell lines, especially in the stem-like subgroup [104]. This treatment induced goblet cell differentiation and reduced cell proliferation, suggesting that forced reduction of DNA methylation diminishes stemness in cancer cells. Although the cancer phenotype is determined by genetic and epigenetic alternations, epigenetic changes have a possible role in regulating cellular differentiation in cancer [105,106].

Thus, epigenetic alteration contributes to define the cancer cell phenotype, and detailed targets for the alteration include the genes that regulate cellular differentiation. Our study found that CDX1, which is considered a key transcriptional factor in intestinal differentiation, is silenced by hypermethylation in its promoter region [104]. However, knockdown of *CDX1* failed to block the induction of goblet cell differentiation in colon cancer cell lines treated with a hypomethylating agent. On the other hand, Ordonez-Moran et al. demonstrated that retinoid therapy leads to cellular differentiation and tumor regression in colonic tumor through the reactivation of *HOXA5* [95], which is a candidate target of DNA hypermethylation in colon tumorigenesis [104]. Interestingly, knockdown of *HOXA5* inhibits cellular differentiation in retinal-treated colon tumors, suggesting that HOXA5 is a candidate master regulator of intestinal differentiation in colon cancer.

## 4. Conclusions

To date, many colorectal CSC marker candidates have been identified. It is reasonable that the various markers reflect the versatile nature of CSCs. Simultaneously, accumulating evidence indicates that both normal and neoplastic cells show bidirectional conversion induced by internal and/or external signals. Currently, stem cells remain at the top of the hierarchy, but some are thought to originate from more highly differentiated subgroups. The bidirectional convertibility of cancer stemness explains why cancer is resistant to therapy, and raises questions about how to detect the true CSC population. Taking intertumoral heterogeneity into account, it is important to find the best combination of CSC and CSC-candidate markers rather than to search for a definitive single CSC marker. In addition, identifying the master regulator of intestinal differentiation in colon cancer is essential for in vivo manipulation of colon cancer phenotype by epigenome editing techniques [107].

**Figure 2:**
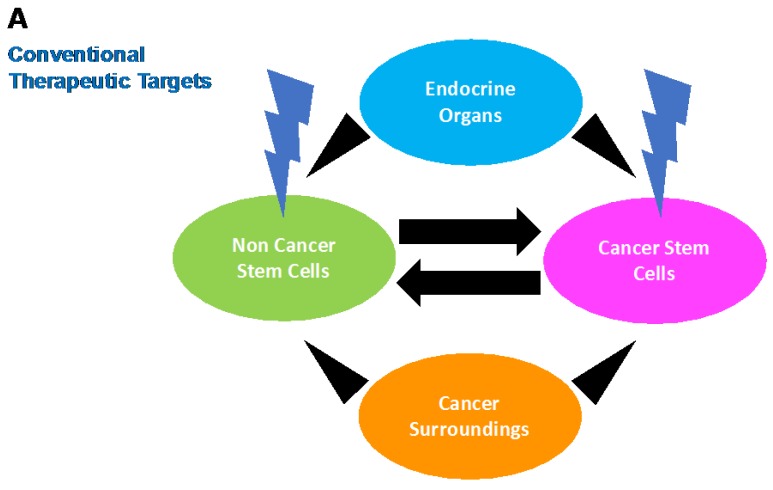

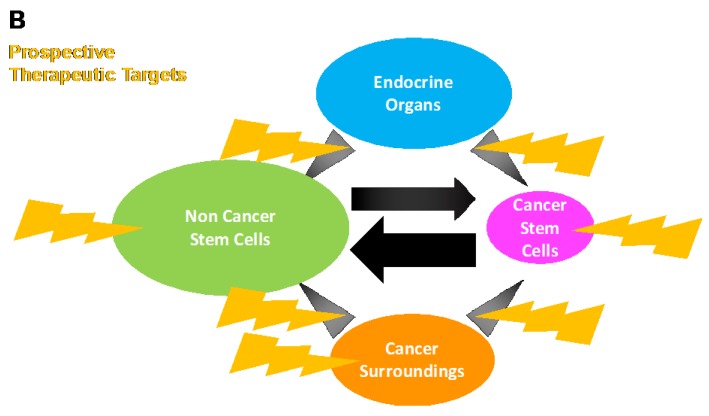
Conventional and prospective targets in cancer therapy: (**A**) Conventional therapy targets exclusively cancer cells without any distinction of cancer stemness. (**B**) On the other hand, prospective therapy involves microenvironment and endocrine status, which induce cancer stemness. Forced shrinking of cancer stem cell population by alteration of external signals is a possible approach for achieving complete response.

In vivo, genetic mutations are not currently correctable, whereas epigenetic changes are treatable. As mentioned above, epigenetic editing is a possible cancer treatment to block the restoration of cancer stemness. In the future, therapeutic targets may be extended to include not only cancer cells but also their microenvironments and endocrine organs that affect the general condition of the patient (Figure 2). Therefore, further research is needed to understand the multifaceted traits of CSCs.
